# The empire of the narrative: Plan making through the prism of classical and postclassical narratologies

**DOI:** 10.1177/14730952221125174

**Published:** 2022-09-16

**Authors:** Laurent Matthey, Julie Ambal, Simon Gaberell, Elena Cogato Lanza

**Affiliations:** 27212 Department of Geography and Environment, University of Geneva, Geneva, Switzerland; 111832 University of Applied Sciences and Arts Western Switzerland[HES-SO]—Geneva, Geneva, Switzerland; Swiss Federal Institute of Technology Lausanne, Lausanne, Switzerland

**Keywords:** narration, plan, signal, narratology, economy of attention, technology of power

## Abstract

This article theorizes the “narrative turn” in urban planning studies, using Gérard Genette’s work to differentiate first- and second-degree narratives. Genette defines the latter as paratexts that determine the public’s reception of the former. The article assesses how second-degree narratives work with different perceptual regimes to construct the reception of the political vision of territory. To that end, it resorts to the recent work of postclassical narratology. Indeed, the latter is particularly interested in the way in which the narrative, in various forms, affects its addressee. Postclassical narratology allows us to renew the theory of narrative in urban planning by focusing on what hypothetically happens in the consciousness of the receiver of the narrative when he or she becomes aware of it. Consequently, the paper sheds light on an emerging aspect of the design process: disambiguating signals embedded in urban planning documents intended for a wider public.

## Introduction

This article draws on contributions that have looked at the narrative dimension of urban planning over the past 30 years (Mandelbaum, 1991; Throgmorton, 1992, 1996, 2003; Sandercock, 2003; Van Hulst, 2012; Ameel, 2016, 2021). It explores how the narration of planning documents has become essential for urban governance, which is increasingly sensitive to creating attention for its actions (Erwein and Matthey, 2019). We first redefine the urban planning narrative in a more restrictive way based on current debates in the field of narratology. Drawing on a distinction made by Gérard Genette (1982 [1992]) between first- and second-degree narratives, we identify two narrative regimes in urban planning.

The first regime is conveyed by technical urban planning documents. These are bearers of a cultural, social, economic, and political vision of a given territory which emit signals primarily to a specific audience, such as technicians and professionals (Hopkins, 2001; Hopkins and Knaap, 2018, 2019). In our opinion, the term narrative is most often used here as a metaphor: while they undeniably convey an imaginary, these documents were not drawn up primarily to tell a story (see also below, page 5).

The second regime transforms the technical document into a more intelligible form for a greater number of people to ensure that it is read according to the plan’s proposed vision. These second-degree narratives communicate the stakes of planning policies and control the reception of the signal embedded in the plan because it is received by a wider audience than planning professionals.

This article discusses second-degree narratives based on a case study of the Geneva Master Plan. It assesses how these accompanying narratives construct the reception of the plan as a signal. Using a conceptual framework from postclassical narratology (Baroni, 2007; 2017), we show that the narrative in urban planning directs the reception of a political vision of the territory by working with different systems of perception and offering cognitive frames. Therefore, this article renews the Foucauldian approach that has often targeted planning discourses as a contribution to the theory of planning. The narration of plans, instead of the discourse, has become essential now. Unlike discourse, narrative has the advantage of rendering the speaker invisible (Benveniste, 1976: 238–241). It therefore takes the recipient into a relationship, inviting them to perceive a story via different points of view. The narrative has become a communication tool, necessary for urban planning documents because their recipients have become more heterogeneous due to the democratization of the plan’s production. New communicational engineering is being instituted to attenuate the noise (in the sense accepted in communication theory) that may accompany the signals a plan emits. In other words, the narrative builds an understanding of the signal.

## A blind task: The role of narrative in an economy of attention

### From prospective fictions to urban planning storytelling

For many years, urban planning research has been interested in the narrative dimension of a practice (planning) that has long been considered from a strictly technical perspective. Researchers have tried to show how urban planning’s transformative power is largely due to its capacity to articulate not only political fictions (Secchi, 1984) but also prospective fictions (Throgmorton, 1996) around material reality. Research has also shown how the narration of the project does or does not favor its realization (Throgmorton, 1992; 1996, 2007), generating more democratic approaches (Mandelbaum, 1991; Forester, 1999; Eckstein and Throgmorton, 2003; Bulkens et al., 2015). Other researchers have explored the structure of plots (Walter, 2013; Keunen and Verraest, 2012) that urban planning narratives develop. They identified styles that can facilitate the understanding and sharing of territorial issues. Drawing on the avant-garde work of feminist geography (Hanson, 1997; Valentine, 2008; McDowell et al., 2014), researchers have endeavored to grasp stylistic processes that guarantee a diversity of voices (Sandercock, 2003, 2010; Eckstein, 2003). Using at times ethnographic methods, monographs have also explained how narratives enable the conception of space (Vitalis and Guéna, 2017; Grigorovschi, 2016). Thus, a number of works have dwelled on the narrative skills necessary to construct a sense of inhabited space (Childs, 2008; Dionne, 2018). Here, it is possible to identify a strong influence from Paul Ricoeur’s writings on the thinking of urban narrative theorists (Uyttenhove et al. 2021): it is the narrative, inasmuch as it makes it possible to give changes meaning, that is considered here.

Monographs devoted to the reception of narratives (Healey, 1996) have also developed due to the growing interest in the narrative dimension of urban planning. Influenced by research in the field of cultural studies (Morley, 1980, 1992) and literary geography (Hones, 2008, 2010, 2011), researchers have examined the effects of context and place on the way that readers appropriate narratives, sometimes attributing to them a completely different meaning. Further, some studies have focused on the use of narrative as a tool of communication by new urban governance (Duranel, 2019; Ouvrard, 2016). This has renewed the line of research that originated particularly in political science, which is centered around discourse (Hastings, 1999; Jacobs, 2006) and its rhetoric (Fisher and Forester, 1993; Fischler, 2000). The narrative is part of what Caune (1997) has called an “aesthetic of communication” that persuades without resorting to argumentation or the agonistic dimension of debate (Hillier, 2003; Rannila and Loivaranta, 2015; Mattila, 2018). Since the early 2000s, researchers have developed themes while working on language and the contributions of Lacanian theory (Gunder, 2003; Hillier, 2003) to the field of “planning through debate” (Healey, 1992). Hillier (2003) showed, for example, the role of Lacanian theory in reconciling Habermasian approaches and intersubjective compromise with agonistic theories of political space in a dialectical transcendence (Hillier, 2003: 55).

Finally, drawing on work done on the aesthetics of communication, some contemporary monographs have focused explicitly on the use of storytelling in urban planning as a tool for good communication. The public authorities’ interest in urban planning storytelling indicates the growing importance of the principles of communicative storytelling that have, hitherto, solely served politics (Matthey, 2014; 2011). These approaches consider the narrative as an instrument that facilitates the exercise of power, from the perspective of the neo-liberal turn of public policies (Purcell, 2009), urban regimes (Irazábal, 2009; Lambelet, 2019), or urban branding (Jensen, 2007).

### Narrating plans to ensure their reception: Channeling attention to reduce the noise of the plan’s emitted signal

The “narrative turn in urban planning” (Ameel, 2021) corresponds to some of the recent planning discourse studies, notably the one by Purcell (2012, 2009). Purcell has shown how neoliberal urban policies use the Habermasian communicative ideal to perpetuate the cultural hegemony of urban powers. However, the transformation of power relations requires radical counter-hegemonic mobilizations (Purcell, 2009) rather than a “communicative turn” (Healey, 1992) in urbanism. Therefore, in the field that is interested in narrative from the perspective of urban power, narrative is perceived differently. Narrative can signify a story that is told by development project owners to promote their acceptance (see Throgmorton’s pioneering work). Further, the narrative approach also perceives narratives by plotting urban planning documents and explaining the narrative modalities that they implement, both graphically and from a more literary perspective. Additionally, research has also been conducted on the tropes used in urban planning documents and how they activate major cultural schemas by mobilizing specific literary genres, such as utopia, idyll, or pastoral (Keunen and Verraest, 2012; Uyttenhove et al., 2021).

Few monographs have developed a narratological approach to urban planning documents that allows us to understand how they mobilize different sensitive registers, and how they affect their recipients. This is probably because narrative approaches have paid more attention to the tropes and other stylistic devices employed in plans and other urban planning documents to tell a story, than to the way that story affects its recipient.

Postclassical narratology opens up a promising avenue here. The term “postclassical narratology” refers to the various approaches that have been developing in the field of narratology since the late 1990s. These approaches, which do not disown but renew classical narratology as it was developed in the 1960s–70s around structuralism (Baroni 2017; Patron 2017; Herman 1997), are more sensitive to the (cultural, historical, etc.) contexts in which narratives emerge. They also look at the diversity of media that are likely to convey narratives. Finally, they are particularly interested in the cognitive and emotional dimension of narratives.

It is in this general context that in the mid-1990s James Phelan began developing a rhetorical approach to narrative by drawing on the intentionality of the one he calls the “teller.” According to Phelan, intentionality needs to be at the center of the analysis (2018: 47). Its primary aim is to understand how the “‘teller’ tries to shape narrative elements to specific ends” (2018: 48). It targets in equal measure the “affective, ethical, as well as esthetic effects of a story.” This rhetorical approach has methodological consequences. Phelan thus seeks to retrospectively reconstruct the effects produced on a recipient by the various narration techniques deployed in a narrative (2018: 48). We will draw on this method further below.

David Hermann (2018) has, for his part, done seminal work on “narrative worldmaking” (2018: 96). He is interested in the “storyworlds” that result from the interaction between the interpretations to which a narrative is subjected, and from the way in which the narrative itself is a producer of meaning. This interest in storyworlds led him to speak of “narrativization practices” (2018: 99). The narrative organizes sometimes heterogeneous events into more or less coherent worlds. In this sense, it is a cognitive resource (2018: 99–100).

Raphael Baroni goes further still in bringing together narratology and cognitive sciences. In his work, *La tension narrative* [“Narrative tension”], he focuses on the aesthetic and affective effect that narrative has on its recipient. To this end, he draws on studies in psychology, particularly cognitive and affective psychology. These make it possible to explain how we are “affected” by narrative structures, whose function is to create suspense or curiosity, for example. As we can see, narrative is no longer approached from the point of view of what classical narratology called the “closure of the text” (i.e., explaining the text by the text, within the text). The focus now is on the recipient of the narrative—and his or her experiences.

Finally, the postclassical current considerably broadens the terrains open to narratology. Thus, in 2004 Marie-Laure Ryan put forward the term “transmedial narratology.” Transmedial narratology postulates that narrative (1) is not limited to literary texts and (2) is deployed through various media (text, image, sound, etc.) and in various ways (books, films, internet, etc.). Seeking to transcend the boundaries of a narratology deemed to stop at language acts alone, postclassical narratology endeavors to detect narrativity in other forms of expression.

This transmedial approach thus leads to a new definition of narrative that is both flexible and rigorous. In the debate between her and David Rudrum (2005; 2006), Marie-Laure Ryan (2006) has suggested classifying acts of language according to their relationship with a prototypical form of narrative (2006: 193), depending on whether they share one, two, three… or all of the nine traits identified (e.g., “Narrative must be about a world populated by individuated existents,” “This world must be situated in history and undergo changes of state”^1^—Ryan, 2006: 193), which attest to *a story’s degree of narrativity*. This open but conditional approach avoids the excesses of another that sees narrative as the result of a social convention, as suggested by David Rudrum. Whatever Rudrum says, narrative does not, Ryan contends, arise from a simple agreement between members of a community about the status of an act of language. Narrative always comes with its own semantics, starting with the inescapable semantics of “telling somebody that something happened” (Phelan quoted by Ryan 2006: 192).

Thus, the approaches promoted by postclassical narratology encourage us to consider the effects that a narrative has on its recipient, particularly from the point of view of its rhetoric. They also invite us to consider how storyworlds are made. They grasp narrative from the point of view of its transmedial manifestations. They are sensitive to different stories’ degree of narrativity. For all these reasons, they are part of renewed approaches to narrative in urban planning which we believe make a contribution to planning theory, particularly from the point of view of the “logic of making plans” (to paraphrase a famous work by Hopkins, 2001). This is where this article’s contribution to planning theory lies: on the one hand, it helps clarify the levels of narrativity of urban planning stories; on the other hand, it attempts to analyze a type of story that is intentionally produced to facilitate, by means of a simulated world, the adoption of a development plan by the general public.

## “What is a narrative in urban planning”: A never-ending story?

Commenting on the diverse notions of narrative used in the field of urban planning, Ameel (2021: 4) observed that early narratological approaches in the field of planning were not very rigorous, often using different terms (story, imagination, and narrative) as synonyms. The acknowledgement of narrative’s omnipresence and polysemy is, of course, not novel. Planning discourse studies reached the same conclusion nearly twenty years ago (Hastings, 1999; Sharp and Richardson, 2001; Jacobs, 2006), insisting on the profusion and diversity of discourses in urban planning. Recently, Ameel (2016, 2021) has identified different uses of narrative in planning (and related areas) before producing a typology. He identified narratives “for, in and of planning.” The first refer to the collective or individual “local narratives” that “precede” and feed into planning. These are distinct from the second type which refer to the narrative found in the “documents or activities” of planning (Ameel, 2021: 13), authored by planners. Finally, “narratives of planning” relate to narratives of planning that has already been conducted.

**Figure 1.**
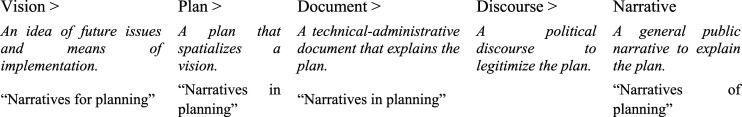
Uses of narrative in planning.

There is another way of considering the diverse uses of narrative in the field of planning, and more specifically in the context of plan making. It consists in looking at them from the point of view of their chronology (Figure 1).

Generally speaking, plan making is understood as a process during which a territorial vision is translated into a plan, technical documents, regulatory apparatuses, and political discourses. Increasingly, these plans, documents, and political discourses are accompanied by explanatory comments aimed at the general public, which come in the form of intentional narrative. Narrative is deployed in different ways in this sequence.

Ryan (2006: 190) insisted that, from a semantic point of view, a narrative is usually an assertion, that is, the most specific register of the narrative mode. Genette believed that, at the very least, there is a narrative when “a thing that has happened” is “recounted” (1983 [2007]: 302–303). However, narrative in urban planning in its ordinary form is most often prescriptive and proceeds from an imperative mode. In a way, it connotes what must or at least should be done. It is deployed in a document that states what should accompany objectives or measures, particularly regarding a plan. From a pragmatic viewpoint, this plan can be understood as a narrative produced by actors, who agree to give it this status (here, we agree with Rudrum). However, from a semantic viewpoint (here, we agree with Ryan), it is not endowed with the attributes of a narrative in the narrower sense.

Of course, while the initial vision is political and strategic in nature, it can nevertheless feed on a narrative imagination. The latter employs what Marie-Laure Ryan (2018) calls “metaphorical narratives.” These are not entirely narrative in nature as they do not meet all the requirements of a story. In other words, their narrativity is weak. They belong more to the realm of imagination, of the “collective values of a culture” (2018: 149). Obviously, this vision can sometimes employ inhabitants’ narratives, which have been collected as part of a participatory approach that feeds into and informs the planning process. Similarly, the plan that spatializes this vision can also make use of weak narrativity. Indeed, plan makers sometimes organize the space into a narrative sequence so as to persuade decision makers.

Rather than getting entangled in an endless epistemological conflict, one could posit that a master plan is a first-degree narrative, that is, narrative in a metaphorical sense. These narratives develop from the narration of a story through techniques—such as tables, graphs, maps, diagrams, action sheets, etc.—that are specific to a discipline (planning or urban planning). Quite regularly, this document uses components of other studies and quotes from interviews or political statements set in boxes or headings: the “objective presence of one text in another” (Genette, 1982 [1992]: 8), which is characteristic of intertextuality, “producing significance” (Riffaterre quoted in Genette, 1982 [1992]: 9) and contributing to the “mechanism[s] of influence” (Bloom quoted in Genette 1982 [1992]: 9).

To satisfy the democratic debate, “second-degree” (1982 [1992]) narratives accompany this first-degree narrative, which they either comment on or complete. These narratives are reworkings of first-degree text, which are more comprehensible to the general public. They also promote the plan’s performativity. These second-degree texts are intentionally narrative in nature. They seek to tell someone about something that has happened, is happening, or will happen (to take up the quote from Phelan already mentioned). They are peopled with individuals to whom things happen. They have a “mental life and react emotionally to the states of the world” (Ryan, 2006: 194). Some of “the events… objectively happen in the story world.” In short, they combine more levels of Ryan’s narrativity scale. In fact, they employ narrative techniques in order to (1) tell the story in a language other than the one used by the experts and (2) bring about a specific effect on the recipient. They allow recipients to appropriate the plan more intuitively, particularly by means of a simulated world. At the same time, they refine the ways in which the plan is incorporated, helping to share a political vision with as many inhabitants as possible.

In fact, their function is often to ensure a reading that conforms to the technical and political vision conveyed by the plan; to emulate a world that conforms to it, employing different perceptive and affectual regimes. From this perspective, these accompanying narratives are part of what many researchers now refer to as an economy of attention (Arpin et al., 2015; Peltola and Tuomisaari, 2015), in the sense that the challenge now is to capture the attention “of a public submerged by proposals… one more attractive than the next” (Citton, 2014). Narrative’s immersive experience constructs the plan’s reception by projecting its recipient into a future world or by making them experience the problems the plan is seeking to solve. Immersive narrative experience reinforces attention (Ash 2012), which is understood as the alignment of certain dispositions (an openness to a certain reality; Ash, 2012).

It is in this respect that postclassical narratological approaches lead us to reconsider the way in which narrative is deployed in planning theory, particularly in relation to plan making. Contemporary (postclassical) narratology increasingly views narratives from the perspective of its effect on the recipient’s sensory apparatus. The narrative creates a “tension” (Baroni, 2007) within its recipient (a “desire to know what happens next”; Bottcher, 2013) and establishes a horizon of expectation. The recipient is immersed in a story. He or she is involved in its understanding. He or she may subscribe to particular perspectives that define the cognitive frame of a given issue. By playing with the narrator’s sensibility, the narrative potentially contributes to these apparatuses currently engaged in the communication of urban projects to reinforce their acceptance (Bailleul, 2008). This strategy is currently implemented in conveying urban planning documents, such as plans. It reduces the noise surrounding a plan’s signal as soon as its recipients are diversified.

This is precisely what we discuss in this study. We postulate that narrative in urban planning is mainly about the narrative of plans. This narration aims to make plans intelligible for a wider audience. It must reduce the “noise” around the emitted signal. This proposal defines a corpus (the narratives that explain the plans) and legitimizes an interest in taking charge of the signal through the narrative.

Below, we will draw on elements from a long-term investigation, which combines old fields devoted to the communicative turn in Geneva’s urban planning (Erwein and Matthey, 2019), and a more recent field centered around the narrative techniques used in Geneva’s contemporary urban planning documents.

## Making narratives speak through second-degree planning: Corpus and method

**Figure 2.**
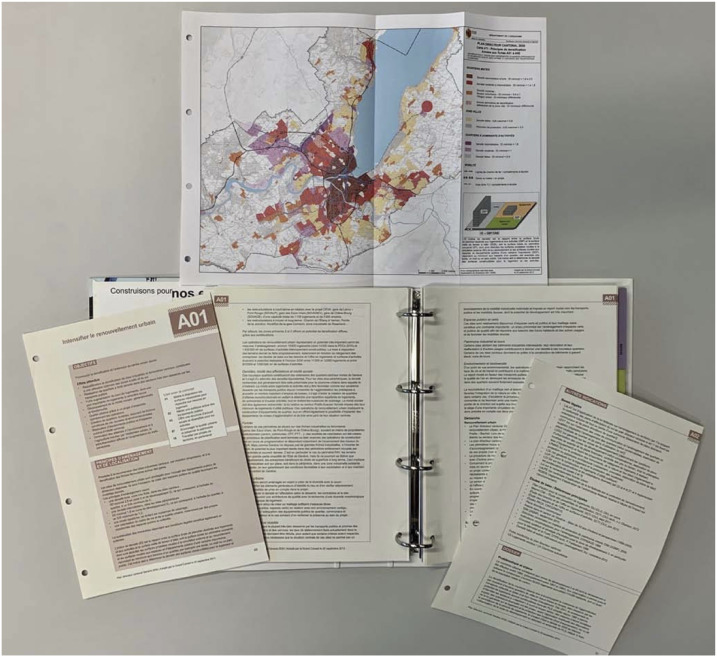
First-degree narrative. In its technical form, the master plan mobilizes a synthesis map (top), action sheets (left), analysis and objectives (center), equal framework, and reference studies (right).

Our analysis focuses on the current transformations of the Geneva 2030 Cantonal Master Plan. In Switzerland, a cantonal master plan is a planning document required by the Federal Law on Spatial Planning (LAT). The aim of this document is to coordinate activities with spatial implications (ARE, 2018). It includes different themes, applicable for 20–25 years. It determines the requirements that must be respected during the planning process: spatial conditions, timetable, and organization (ARE, 2018). The whole plan takes the form of a technical document that lists objectives, associates them with measures, and establishes the scope of action (Figure 2). This constitutes the first-level narrative, which is primarily intended for professionals.

The federal government must approve each of the 26 cantonal master plans after lengthy procedures. The first version of the plan is presented to the civil society (especially non-governmental and professional associations) and municipalities for public consultation. This results in a number of comments that are eventually addressed by the technical services of the public administration. The revised document is then submitted to the cantonal parliament, which accepts or rejects it via a vote. Further, the plan is submitted to the Federal Office for Spatial Development, which verifies its conformity with current laws, particularly those concerned with planning objectives, and accepts it either completely or partially with requested revisions. Finally, the plan is forwarded to the federal executive, where the document is ratified. If it is not ratified, then the canton’s spatial development is blocked.

Increasingly, this document, which determines a community’s future for the following 20–25 years, has triggered strong debates among both citizens and politicians. Let us take, for example, the Geneva 2030 Cantonal Master Plan, which was submitted for public inquiry in the context of a severe housing crisis (the vacancy rate was 0.15%, far from the 1.5% synonymous with a fluid market in Switzerland) caused by very strong economic and demographic growth. Approximately 544 comments from the public inquiry conducted in 2011 were referred to the technical services. The municipalities also had reservations because they were reluctant to welcome new urbanization projects in their region. Therefore, there was a strong possibility that the parliament would reject the document. The cantonal administration noted the need for significant “educational work” (framework, cantonal administration), which had to be done in relation to both elected municipal representatives and all the inhabitants of the canton. The aim was to make people understand the challenges of a cross-border region (Greater Geneva), where the central unit (the canton of Geneva) is an employment hub, while the peripheral areas (France and the canton of Vaud) specialize in housing. At the same time, one had to bear in mind the Franco–Swiss territorial pact signed by the partners of Greater Geneva. Geneva produced housing while measures were being promoted to rebalance the region and stimulate the creation of activities in France.

Consequently, the need for a pedagogical approach led to the creation of a “general public section,” the *Genève, Envie* [“Geneva, Desire”] brochure, in the Geneva 2030 Cantonal Master Plan’s final version. It “presents the issues and objectives of the master plan”:

This document has been designed to allow a non-specialist audience to understand the underlying objectives of the master plan, which is very technical in its writing and form. This section has not been updated. The brochure also includes a simplified map of the developments proposed by the cantonal master plan and a brief history of urban planning in Geneva.

**Figure 3.**
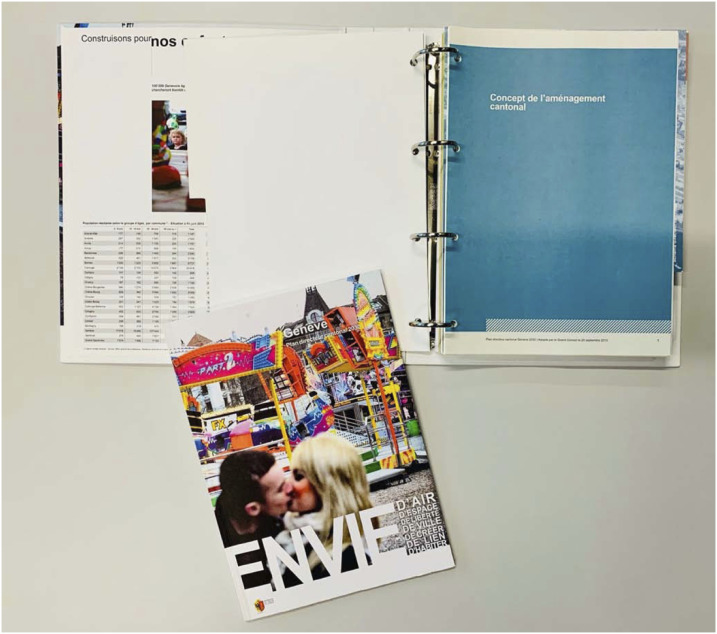
Peritext: Framing the narrative. Final version of the 2030 Master Plan of the Republic and Canton of Geneva along with other technical documents. Below is a detachable booklet for the public.

This brochure (and its by-products) is the subject of our narratological interpretation that assesses the techniques used to direct the audience’s attention. The brochure is an example of the “echo” narrative from Genette’s typology.^2^ It is a narrative that mobilizes, sometimes integrates, and above all comments on the “first-degree narrative” of the master plan. This narrative is called paratextuality; its function is to anticipate the public’s reception of a technical document (a master plan, a neighborhood plan, etc.). At the same time, the second-degree narrative is a political option that addresses urban problems. Figure 3

**Figure 4.**
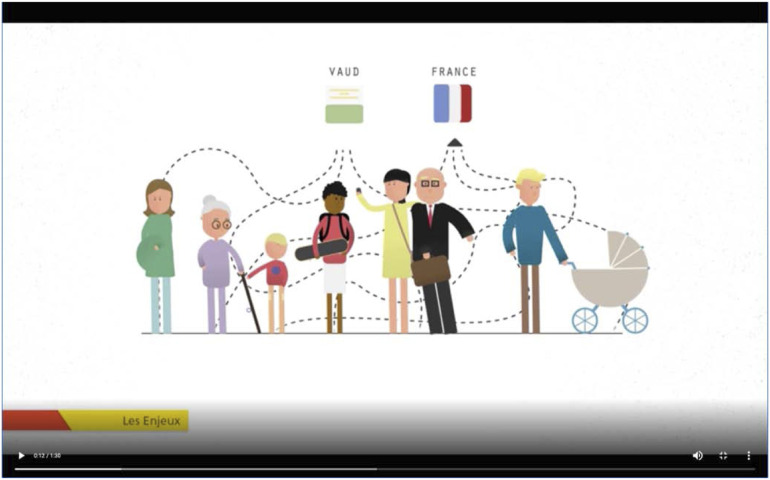
Epitext: Disseminating the narrative. Screenshot of a clip about the issues at stake in the Geneva region. Commentary: “Today, thousands of Genevans cannot find housing. To avoid their leaving the canton and increased commuting throughout the area, we need to develop places for living, working, and recreation.”

**Figure 5.**
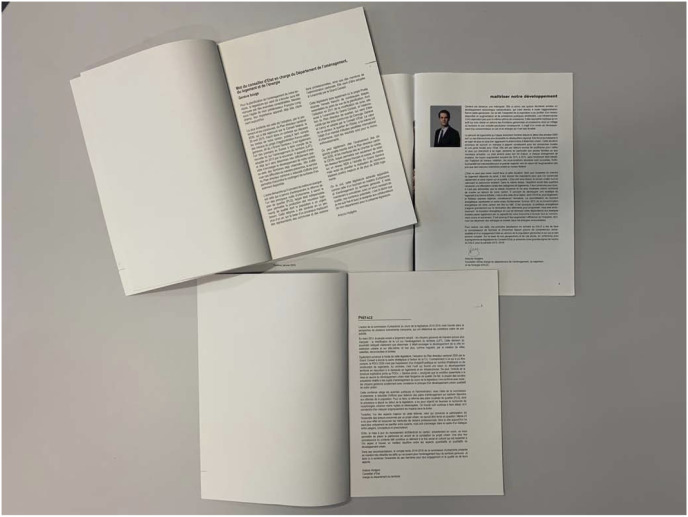
Metatext: Overlaying first-degree narrative. The use of remote accompanying texts of the 2030 Master Plan of the Republic and Canton of Geneva: the prefaces to the thirteenth (top left) and fourteenth (bottom) Reports of the Planning Commission (Geneva, 2010–2014) and the 2015–2018 Roadmap of the Department of Planning, Housing, and Energy.

It should be noted that this paratext can take on various forms. It can be directly linked to the text that it comments on as a preface, annotations, text box. It can also be deployed remotely via other media or communication supports (Genette, 1992 [1982]: 11). For example, a promotional film, an exhibition, an elected official’s speech (Figures 4 and 5).

These often implicit commentary relationships between different texts provide a capacity for dissemination that makes the narrative an interesting tool for communicating urban policies. First-degree texts are covered with a thick layer of glosses (Figure 5), which are at the center of our narratological investigation. The analysis we offer mobilizes elements from a didactic film and accompanying texts signed by the elected officials who are in charge of Genevan territorial planning. All these documents, except the Geneva 2030 Cantonal Master Plan (in its strict sense), are second-degree narratives. Their aim is to explain how an interested but uninformed public should understand the signals emitted by the plan in a language other than that used in the technical documents.

For the purposes of our analysis, we incorporated elements from postclassical narratology (Herman, 1997; Baroni, 2007, 2017; Baetens, 2017) that shed light on the cognitive framing which operates through the story’s narration while producing the urban planning narrative. We will reconstruct our material taking our inspiration from the principles of hermeneutic description (Laplantine, 1996), setting out our interpretations at the same time as we describe our corpus (Revel, 1995).

## Story/narration/narrative: Creating tension among the addressees

Second-degree narratives are produced by organizing a story using narrative techniques. The tension identified by Genette (1972 [2007]: 11–20) between the story (what is being told), the narrative (the way the story is being told), and narration (the exposition choices that structure the story) can be found here.

Each of these constituents of what Genette called the “narrative fact”^3^ (1983 [2007]: 297) is open to a specific analysis and refers to a particular reception of the text produced. Choosing the story to be told, its plotting, and the resulting narrative are three distinct but complementary moments.

### Story

If we try to rephrase the purpose of technical documents (first-degree narrative, in this case the 2030 Master Plan of the Republic and Canton of Geneva) using layperson’s language, the following story emerges: a public authority is required to produce 50,000 housing units by 2030 to accommodate the strong economic and demographic growth predicted by various models. The region in question is one of the most dynamic and attractive regions in Europe (UBS, 2021).

In fact, creating 50,000 housing units must avoid an exodus of Genevans to the canton of Vaud (the district of Nyon, in particular) along the French border, where housing is more abundant and less expensive. Importantly, the story that this type of technical document develops is not always the same (Joye and Kaufmann, 1998; Léveillé, 2011). Plans from 1945 to 1949 expressed the necessity to modernize an area that had become international (Canosa et al., 2003). Plans during the 1960s told the story of an area that had conquered its periphery but was concerned about the quality of life (Canosa et al., 2003). History mentions a plot in relation to the region at a given moment in its political, economic, social, and environmental future. Now, this plot goes through a certain number of stages that, from an initial but now-broken equilibrium, moves toward a new stable equilibrium.

### Plotting

The narrative becomes that of a personified territory (giving Geneva a protean character). It has known from time immemorial how to “provide intelligent and continuous proximity between built and unbuilt areas,” developing like a “starfish” along “historical axes of communication” (République et canton de Genève, 2013: 4). However, this balance is now under threat: “like a starfish, it would not be good if some of its arms were to atrophy, while others were to blister” (République et canton de Genève, 2013: 4). Will Geneva manage to preserve this balance? Various adventures unfold, with just as many chapters and corresponding desires. The brochure *Genève, Envie* includes eight chapters^4^ and offers a narrative and pictorial summary of the challenges presented in the technical document.

Sometimes, the plot’s resolution is deferred, at other times, the quest is fulfilled. Opponents and helpers mobilize to maintain the addressee’s attention, but also educate him or her as they determine the rights and wrongs of planning. The story’s addressee is progressively intrigued by the future of Geneva and the starfish, being less interested in reading the technical document’s objectives and measurement sheets.

### Narrating

Narration refers to the engineering (the “mechanisms of the text,” its “mechanical” aspect—Genette 1983 [2007]: 294) that produces a narrative. Narration is that aspect of narratology that Genette refers to as “formal” or “modal” (Genette 1983 [2007]: 300). It analyzes the narrative as a mode of “representing stories” (Genette 1983 [2007]: 300) because it focuses on “figures,” that is, narrative procedures. Analysis of these processes facilitates the understanding of the “organization of the narrative” (Guillemette and Lévesque, 2016) based on questions of voice, mode, and time. The narrative unfolds from a speaker (voice), adopts a perspective (mode), and plays a role in exposure times (time). Subsequently, it maintains strong links with the story’s construction, meaning it is likely to “have a direct influence on the dynamics of the plot” (Baroni, 2017: 82). Narrative devices are, indeed, “textual means deployed by the author to … tie up or untie a plot, arouse suspense, inspire curiosity or surprise the reader” (Baroni, 2017: 83). Narration arouses “curiosity” or “suspense” as it delays information or reveals too much information. Slowly, it draws the recipient into an immersive universe, offering various thematic frames and frames of experience.

In the brochure *Genève, Envie*, the narrative begins in the middle of things (*in media res*, as narratologists would say), immediately plunging the addressee into the context of an action. The plot revolves around a disequilibrium between jobs, inhabitants, and housing. The following is a comment by an external narrator on this action.

One hundred thousand Genevans are now under the age of 20. They are the ones that the authorities of this precious region must think about when painting, in broad strokes, the future of our spaces. To avoid their being forced into exile tomorrow, we must offer them places where they can live, work, and enjoy themselves (République et canton de Genève, 2013: 2).

A threat persists that is accentuated in the next paragraph: “Where and how will our children live?” As the story progresses, the narrator describes the scene of the action (an idyllic place: “Geneva has a particularly rich green belt around its urban areas”), introduces its characters (Geneva, the protean territory, the Genevans, the commuters, the tourists, the delinquents, the young, the families, the elderly, and the taxpayers), and plays with the idea of time (everything that Geneva has inherited and all that it has to accomplish). The perspective changes because the territory becomes a character in the story until the narrator provides an overview. Sometimes the narration dwells on details, such as numerical descriptions, that are meant to inform and educate the reader: “parks and green areas … cover 2.5% [of the region]. Housing is concentrated in 23% of the cantonal area, almost half of which is in the villa zone” (République et canton de Genève, 2013: 4). The reader may prefer to skip these passages, but they need this content to discover how the story ends; it seems reading secondary narratives is not as dry as technical documents.

### Narrative

Finally, the narrative results from the story’s narration (its “product”; Genette 1983 [2007]: 298). It is a story which is established through a plot and related using various techniques. It requires engineering. For those wishing to understand the renewal of technologies, which facilitates the circulation of power over the city, the process makes the analysis interesting. Analyzing the narrative, taking an interest in the “workings of the plot” (Baroni, 2017), the way in which the narrative establishes “narrative tension” (Baroni, 2017), allows access to the intentions or auctorial stance of the author(s). Thanks to singular techniques, the addressee is affected, the reader may or may not identify with a narrator, the addressee may adopt the narrator’s perspective, and the addressee may begin to think according to a particular schema. The explicitness of the narrative’s message is one lever among others in the analysis of urban powers. The following section discusses the level of narration more systematically.

## Voice, view, rhythm, value: Engineering through attention

Understanding urban-planning narrative as a technology of power requires an examination of the engineering implemented to create the effect desired. The recent update of the instrument of classical narratology to postclassical narratology invites interesting avenues for urban studies. Postclassical narratology has renewed narrative analysis as it focuses not only on the mechanisms of the narrative, but also on the influence it has on the recipient. Therefore, there is an opportunity to identify a series of methodological principles in narration that explain how it affects the addressee.

### Giving a voice and framing a perspective

The first principle states that a voice always communicates the narrative and mobilizes one or more perspectives. The question of voice and perspectives has given rise to numerous works in narratology. First, it produced an interest in identifying different types of narrators according to their “relationship to the story” or the narrative “level”^5^ (Jouve, 1999 [2017]). Such research identified various functions of the narrator, depending on whether they narrated, organized the text, or communicated additional elements necessary for understanding the plot. Later, it was established that voice cannot be reduced to perspective. François Jost (quoted in Baroni, 2017: 99) proposed distinguishing between focalization and “ocularization/auricularization,” that is, between the information stated by the narrator and information that the audience gets through the characters’ sensations. Subsequently, others have identified the need to differentiate between the “voice that narrates … [and] consciousness that perceives” (Jouve, 1999 [2017]).

Therefore, a narrative mobilizes a “perceptual or cognitive focus.” The “management of information that makes it possible to understand a narrative situation” (Baroni, 2017: 100) underpins it. Sometimes, a narrative comes from an external and objective perspective that states facts, lists problems, and describes solutions (when the master plan is considered a technical document). Other times, it comes from a narrator who is also the author (e.g., the testimony of an inhabitant). It may also originate from an omniscient narrator who orchestrates different perspectives (the narrative of the great project grasped by the communicators). Finally, the author sometimes plays on the confusion between author and narrator or becomes a character of its narrative (e.g., the narrative of a project developed by a designer during an urban design competition).

Each of these foci causes a specific reception because it plays on the recipient’s space. This causes the addressee to frame the plot in an expected way. Therefore, identifying instances of narrative fiction and analyzing the relationships that may exist between the instances of fiction and real people become a possible lever of critical hermeneutics relevant to urban planning. For example, a close analysis of the relationships that are woven between the narrator and recipient allows the researcher to sketch the recipient’s contours, understand what this reader is supposed to know, what the reader is likely to be sensitive toward, and what touches and moves the reader. The recipient of the *Genève, Envie* brochure is a middle-class urbanite. They are attached to Geneva, knowing its implicit cultural and economic history. They have difficulty finding a home that matches their residential aspirations. They do not question the region’s economic attractiveness. Life in suburban areas appears to be the worst, unless a polycentric model is developed.

### Pacing

The second principle signifies that the narrative always carries out work on time. This necessitates an analysis of the “relations between the time of the story (measurable in centuries, years, days, hours, etc.) and the time of the narrative (measurable in numbers of lines or pages),” that is, the “time told” and the “time taken to tell” (Jouve, 1999 [2017]: 43). Different questions have allowed narratologists to capture various uses of time in a narrative. Generally, these questions seek to understand “when” and “at what pace” a story is told, and “how often” and in “what order” the events are narrated.

In urban planning, the narrative focuses on narrating future history (a world organized around the principle of a “compact, multipolar, and green Geneva” [République et canton de Genève, 2013: 8]), a near or distant past (a Geneva that has preserved nearly 50% of its area as an agricultural zone), or a contemporary event (a Geneva without housing, a situation that is driving out its children). The narrative can linger on certain details that the narrator, but not necessarily the reader, finds important (providing figures and recounting all the steps in an embedded narrative that sets out a master plan). It can also use ellipses, speeding up certain sequences that the reader would perhaps like to see developed to understand better (it is satisfied with announcing the scope of major projects rather than explaining how each scope was determined). The narrative can recount the same event one or more times from different perspectives (from the perspective of an omniscient narrator and then from that of a character with limited rationality), be told by different narrators (a resident’s perspective may differ from that of an elected official or an investor), or use different modes of narration (an event can be recounted in the text or in the form of boxes, headings, or images). Finally, the order in which the constituent events of a story are told is a powerful storytelling agent. The story can begin with the end (“Today, thousands of Genevans cannot find housing”) or go back to the causes (lack of “places for living, working, and recreation”; République canton de Genève, 2018). It can follow an external and objective chronology that suggests a long and inevitable decline.

The spirit of the laws originally intended to protect citizens from the effects of excessive rent variations has been perverted. Low rents do not necessarily benefit people on low incomes. … young households, working families, are the first to suffer from this evolution of things. (Republique et canton de Genève, 2013: 18–19)

This can only be salvaged by the solutions promoted by a development plan: “to house the 100,000 children currently residing in Geneva, we need to build sufficient housing over the next 20 years” (Republique et canton de Genève, 2013: 22).

In short, the time taken to narrate a plot’s sequences signifies attentional pace that a narrative encourages. This information makes it possible to understand what the narrator considers useful for the addressee. However, the narrative does more than direct attention; rather, it organizes a hierarchy of territorial values.

### The “differential valence”^6^ of perspectives

The third principle signifies that the narrative organizes values. The early narrative semiotics put forward different models (Proppian model, actancial model, etc.) offering a pathway to the “vision,” “values,” and “intention” (Jouve, 1999 [2017]: 64) embedded in a narrative. In a narrative, some “actants” hinder the plot’s resolution (the opponents) and thwart the quest, while others promote resolution (the helpers). These actants are often associated with positive or negative values. Focusing on their characterization facilitates the understanding of the system of values and their meaning conveyed by the narrative. According to narratology, it is up to “thematic analysis” to understand the “preferential axes” (Jouve, 1999 [2017]: 64) mobilized by a narrative and the fields it prioritizes. Similarly, interest in the “narrative grammar” or “narrative program” that fuels a narrative allows dealing with its “value effect,” that is, the way it “conveys an ideology and transmits it” (Jouve, 1999 [2017]: 64).

The principle of a “compact, multipolar, and green urban area” in the Geneva Constitution becomes, in the *Genève, Envie* brochure, the sign of a “desire for the city” animating the local population (République et canton de Genève, 2013: 8). Compactness, a helper which should facilitate the arduous task of creating 50,000 housing units by 2030 while preserving the cramped region’s countryside, is then associated with “what is best in humans”: “civility, urbanity, city, citizenship” (République et canton de Genève, 2013: 8). Multipolarity, another helper that balances the Genevan territory, prevents its center from being “deserted at night and on weekends, except for a few tourists and illicit activities.” It preserves inhabitants from an “absurd daily migration on asphyxiated transport routes between center and suburbs—these banished places, these non-places between town and country” (République et canton de Genève, 2013: 8). A twilight world, which is the old model of an active center and a periphery designed only for sleeping, emerges in the background. It characterizes the values associated with the reversal of the proposals of the Master Plan of the Republic and Canton of Geneva. The narrative opponents are individual housing, mobility through cars, and residing on the periphery of the Genevan territorial system. In another narrative, these narrative opponents could have been described as rural idyll locations for residences outside the city.

In fact, analyzing the differential valence of perspectives is essential because the narrative, which aims to create narrative tension in order to develop a plot and arouse curiosity or build suspense, seeks to be immersive. The addressee is immersed in the plot; the addressee’s attention follows the proposed cognitive schema; the addressee gradually moves toward a possible world rather than in a different direction as a result of the different choices made by the author or auctorial authority. Therefore, second-degree narratives in urban planning can be seen as mechanisms that aim to draw attention and open new fields for planning theory, more specifically, the critical analysis of immersive urban communication devices.

## Making second-degree urban planning narratives talk

This contribution focuses on second-degree narratives of planning documents and how they are part of an economy of attention aimed at identifying a specific narrative regime. These narratives are particularly revealing as they widen the circle of addressees and frame the reception of technical documents. We have thus observed the processes of defining a story, setting up a plot, and narrating the story, which are based on intention. These narrative techniques used during narrations provide access to cognitive framing strategies, create tension among the addressees, and construct a horizon of expectation that, to our knowledge, can facilitate analysis in the field of planning. Therefore, the approach proposed in this article is likely to shed new light on the numerous studies that have been interested in planning discourse as a modality for both the exercise of power and neo-liberal marketing. More significantly, this approach allows for a better understanding of certain contemporary aspects that participate in producing plans. The appearance of second-degree narratives is a way of reducing the noise of signals emitted by first-degree documents.

Second-degree narratives are more than just narratively stated speech. They are also more than communication narratives. Discourse lies within the space of rhetoric. It aims to persuade by using codified figures of speech, in a space that remains inter-subjective. Such narratives tend to forget the political space of debate by immersing the addressee or narrator into the story. Symmetrically, the communication narrative invites the addressee to experience content through a story with a meaningful moral. A reading pact, stated at the narrative’s beginning (I will start by telling you a story), generally identifies the simulation that is offered to the addressee. As we have tried to show, second-degree narratives often proceed by creating an ellipsis of the reading pact, which is often rendered implicit. The planning document narrative does not say that it is a narrative. It tells a lived reality from a given point of view, usually that of a narrator. It diminishes the possibility of a debate.

Narratives in urban planning can be understood as a tool for those who wish to extend the project of the archaeology of power (Foucault, 1976). Second-degree narratives shape the reception of information and direct the addressee’s attention toward one spatio-temporal dimension as opposed to another. At the same time, they provide a cognitive framework for addressees. Consequently, the use of narrative in urban planning becomes a new technology of power, this deduction being in line with some of the conclusions that propose the deployment of a Foucauldian apparatus to analyze planning discourses (Fischler, 2000).

The main challenge, however, is to use this apparatus to analyze the narratives that city builders produce in order to support a pedagogy surrounding a project. Second-degree narratives are a tool used to shape the reception of urban projects as desired by local authorities (Lambelet, 2019).

## Notes

1. The nine defining features of a narrative according to Ryan (2006: 193) are: “[1] Narrative must be about a world populated by individuated existents; [2] This world must be situated in history and undergo changes of state; [3] The changes of state must be caused be external events, not by natural evolution (such as aging); [4] Some of the participants in the events must be human or anthropomorphic agents who have a mental life and react emotionally to the states of the world; [5] Some of the events must be purposeful actions by these agents, that is, the agents must be motivated by conflicts and their deeds must be aimed toward the solving of problems; [6] The sequence of events must form a unified causal chain and lead to closure; [7] Some of the events must be non-habitual; [8] The events must objectively happen in the storyworld; [9] The story must have a point.”

2. For Gérard Genette (1992 [1982]), “echo” texts are those whose function is to comment on other texts in order to make them more intelligible.

3. Although for Gérard Genette (1983 [1997]) the narrative fact relates to the story/narration/narrative tripartition, other researchers have shown that the narrative fact transcends these strict narratological boundaries (Marti, 2017) since it undoubtedly refers to cognitive schemas (Schaeffer, 2020: 15) which lead to information being organized into a logical sequence of events.

4. “Geneva, desire,” “desire for space,” “desire for city,” “desire to create,” “desire to live,” “desire for freedom,” “desire for bonds.” The technical document has seven sections: “Cantonal development concept,” “Cantonal master plan,” “Basic studies and master planning,” “Glossary,” “List of abbreviations,” “Cartographic annexes,” and “Master Plan map.”

5. Vincent Jouve distinguishes three levels in the analysis of narratives. The discursive level looks at the way in which a narrative is organized from the point of view of its narrator. The narrative level examines the sequence of events. And the programmatic level seeks to understand how a narratee’s attention is directed by the narrative.

6. We have borrowed the phrase from François Héritier (1981), who refers to the “differential valence of the sexes.”
